# P-438. Microbiology and Epidemiology of Lemierre’s Syndrome in the U.S. Military Health System

**DOI:** 10.1093/ofid/ofaf695.653

**Published:** 2026-01-11

**Authors:** Erinne Salo, Sarah Deperrior, Milissa U Jones, Daniel J Adams

**Affiliations:** Naval Medical Center Portsmouth, Suffolk, Virginia; Defense Centers for Public Health, Portsmouth, Virginia; Uniformed Services University, Bethesda, Maryland; Uniformed Services University of the Health Sciences, Chesapeake, Virginia

## Abstract

**Background:**

Lemierre’s Syndrome (LS), or septic thrombophlebitis of the internal jugular vein, is a rare complication of oropharyngeal infections. Given the severity of this infection and its nonspecific clinical presentation, accurate microbiologic data are essential to guide optimal empiric antibiotic therapy. *Fusobacterium necrophorum* has been classically implicated in LS; however, polymicrobial infections and other pathogens, including *S. aureus*, are increasingly identified. The objective of this analysis is to clarify the microbiology and epidemiology of LS specifically in pediatric and young adult populations within the U.S. Military Health System.

**Table 1.**
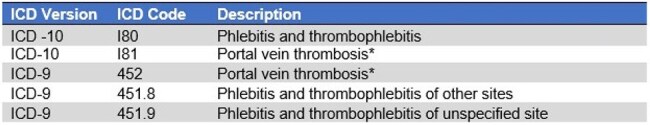
ICD-9 and ICD-10 codes used to identify patients with thrombophlebitis

**Table 2.**
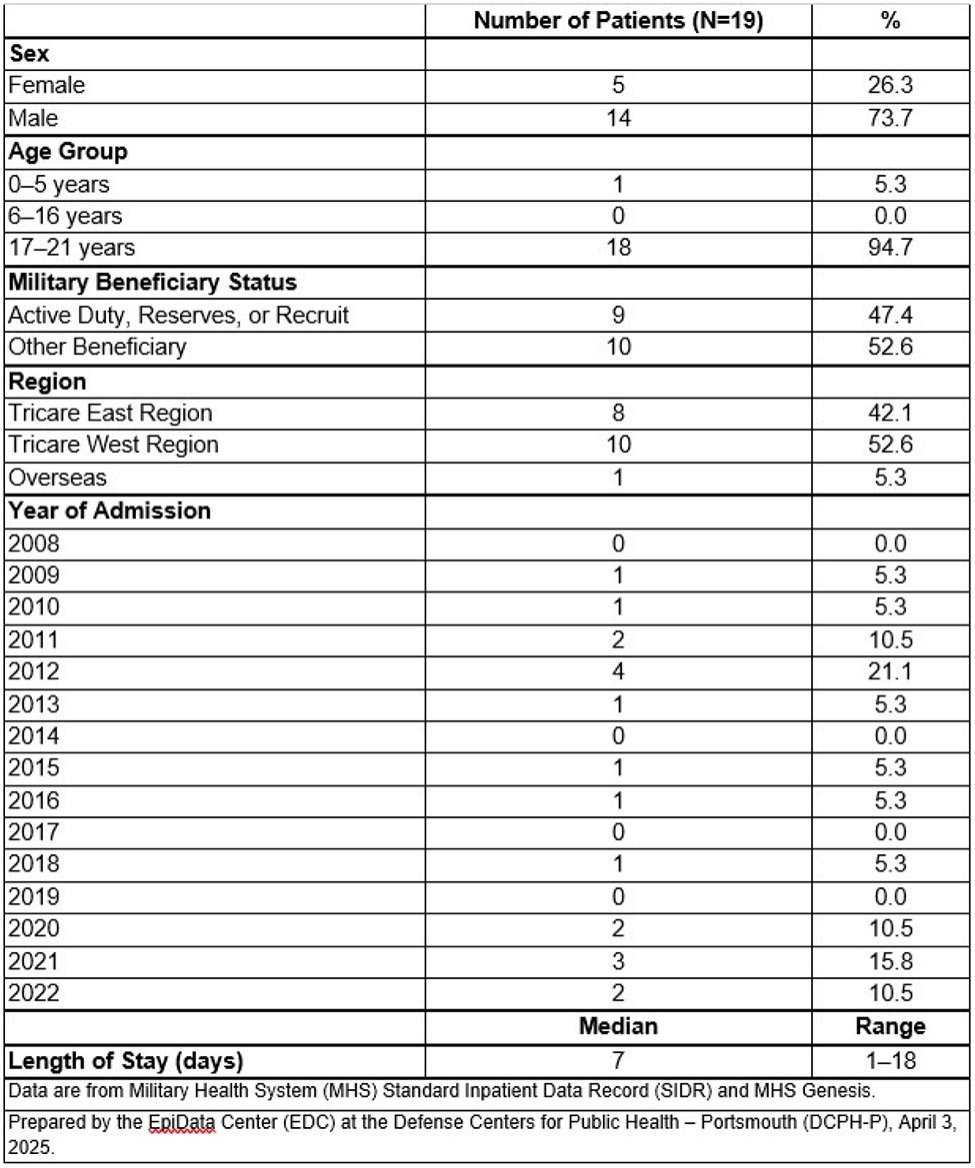
Demographic data for patients hospitalized with Lemierre's Syndrome

**Methods:**

We performed a retrospective case series of Department of Defense beneficiaries with LS. Patients under the age of 22 years admitted to any Military Treatment Facility between October 1, 2008 and September 30, 2022, with ICD-9/10 diagnosis codes for thrombophlebitis (Table 1) were identified. Microbiology and radiology records were then used to exclude patients without either a head/neck vein thrombus or *Fusobacterium* growth on blood culture for greater specificity of capturing LS cases. Demographic data on age, sex, region, beneficiary status and admission year were also collected.

**Figure 1.**
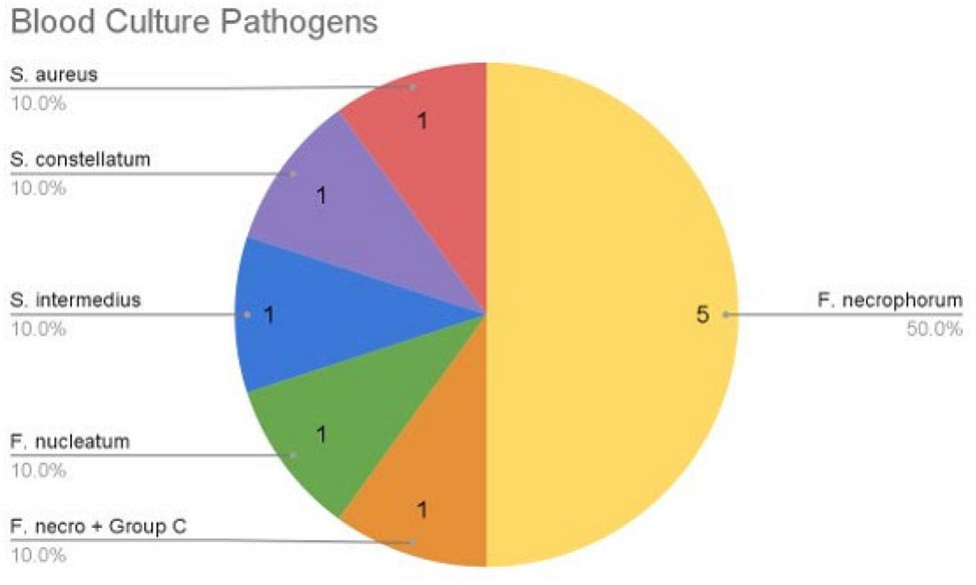
Bacteria isolated from blood cultures of patients with Lemierre’s Syndrome

**Results:**

We identified 19 patients with LS. All but 1 were between 17 and 21 years of age; 74% were male; and 47% were active-duty service members (Table 2). Positive blood cultures were present in 53% of LS cases (Figure 1). *Fusobacterium* species were the most isolated LS bloodstream pathogens (70% of cases). The remaining culture data showed 1 (10%) *S. aureus,* 1 (10%) *S. intermedius,* and 1 (10%) *S. constellatus* infection. One patient had a polymicrobial LS infection (*Fusobacterium* + Group C *Streptococcus).*

**Conclusion:**

Lemierre’s Syndrome in the U.S. Military Health System predominantly occurs in adolescent males with a causative pathogen identified in approximately 53% of cases. *Fusobacterium* species, especially *F. necrophorum*, are the leading LS pathogens; *Staphylococcal* and *Streptococcal* species are isolated less frequently, or as *Fusobacterium* co-pathogens. These findings suggest that beta-lactam-beta-lactamase inhibitor combination antibiotics or ceftriaxone with metronidazole remain optimal empiric therapies for treating LS.

**Disclosures:**

All Authors: No reported disclosures

